# Editorial: Mobile Genetic Elements in Cellular Differentiation, Genome Stability, and Cancer

**DOI:** 10.3389/fchem.2017.00108

**Published:** 2017-12-04

**Authors:** Tammy A. Morrish, Jose L. Garcia-Pérez

**Affiliations:** ^1^Independent Researcher, Ann Arbor, MI, United States; ^2^MRC Human Genetics Unit, Institute of Genetics and Molecular Medicine (IGMM), University of Edinburgh, Edinburgh, United Kingdom; ^3^Junta de Andalucía de Genómica e Investigación Oncológica (GENYO), Granada, Spain; ^4^Centre for Genomics and Oncological Research, University of Granada, Granada, Spain

**Keywords:** mobile DNA, reverse transcriptase, genome stability, cellular differentiation, model organisms, retrotransposon, transposon, DNA repair

The human genome, as well as the genome of most organisms, harbors various types and abundances of transposable element derived repeats (Lander, 2001; Waterston et al., 2002). The topic on: “Mobile Genetic Elements in Cellular Differentiation, Genome Stability, and Cancer,” includes a collection of original research articles and reviews, which address the impact of reverse transcriptases, including the ones coded by transposable elements, on both basic biological mechanisms and disease. In 1970, the discovery of reverse transcriptases or RNA-dependent DNA polymerases, was reported by two different laboratories (Baltimore, 1970; Temin and Mizutani, 1970). Since then numerous studies regarding retroviral reverse transcriptases have significantly contributed to the characterization and biology of may different retrovirus and retroelements. These studies continue to be of interest for the prevention and treatment of various retroviral induced human diseases and for the basic understanding of the origin of retroviruses. In addition the knowledge of reverse transcription has been harnessed for basic use in molecular biology and other applications, including recent widely used methods such as RNAseq. As retroviruses are considered exogenously derived reverse transcriptases, the subsequent discovery in 1987 of telomerase, also considered an endogenous RNA-dependent DNA polymerase, has significantly contributed to the understanding of one of the predominant mechanisms of telomere maintenance that contributes to most, but not all organisms with linear chromosomes (Greider and Blackburn, 1985; Biessmann et al., 1990). Yet, sequences encoding for endogenous RNA-dependent DNA polymerases are not limited to telomerase. The isolation and subsequent genetic, biochemical, and molecular characterization of human full-length non-Long Terminal Repeat (LTR) retrotransposons, termed **L**ong **In**terspersed **E**lements (LINE-1) demonstrated that elements formally encode a reverse transcriptase activity (Dombroski et al., 1991; Mathias et al., 1991; Feng et al., 1996; Moran et al., 1996). Non-LTR retrotransposons are not limited to the human genome, and are present as full-length and/or truncated, rearranged, inactive remnants in many other genomes. In addition, the reverse transcriptase activities encoded by non-LTR retrotransposons share sequence identity with many other reverse transcriptases (Nakamura et al., 1997; Malik et al., 1999). Furthermore, non-LTR retrotransposons rely on the encoded reverse transcriptase for integration, typically by target-primed reverse transcription (TPRT), which was initially biochemically defined using the non-LTR retrotransposon R2Bm, from *Bombyx mori* (Luan et al., 1993). A review by Onozawa and Aplan included in this topic, describes two different types of LINE-1 reverse transcriptase-mediated template sequence insertion polymorphisms (TSIPs), or integration structures that are polymorphic in the human genome (Onozawa and Aplan). The characteristics of class2 structures allude to the occurrence of additional integration mechanisms by the LINE-1 reverse transcriptase that may occur in germ cells or during embryogenesis (Onozawa and Aplan). To note, the features described in these class2 structures are consistent with previous reports of endonuclease-independent LINE-1 retrotransposition (Eickbush, 2002; Morrish et al., 2002).

Phylogenetic analysis of the reverse transcriptase domains support the idea that retroviruses and telomerase evolved from non-LTR retrotransposons, due to the gain or loss of LTR sequence and/or sequences encoding for specific domains (Xiong and Eickbush, 1988; Malik et al., 1999). These early phylogenetic studies are consistent with the protovirus hypothesis proposed by Temin, that (1) retroviruses are likely derived from endogenous retrotransposons and (2) mutations that arise due to the mobility of retrotransposons could potentially activate oncogenes or inactivate tumor suppressor genes, perhaps contributing to tumorigenesis (Temin, 1971; Shimotohno et al., 1980). As LINE-1 elements are active in tumors, yet transcriptionally repressed in many somatic cell types, there was much interest to understand the extent that LINE-1 retrotransposition contributes to tumorigenesis (Solyom et al., 2012; Shukla et al., 2013; Doucet-O'Hare et al., 2015; Ewing et al., 2015; Rodic et al., 2015). Included in this topic is original research using bioinformatic approaches to examine LINE-1 expression and insertion profiles using RNA-seq data from normal and primary tumor samples collected using the Cancer Genome Atlas (TCGA) (Clayton et al.). Here the authors examined the expression and integration differences in breast invasive carcinoma, head and neck squamous carcinoma, and lung adenocarcinoma and their analysis indicates two cases of LINE-1 mediated insertions near two different tumor suppressor genes, including an Alu insertion into the *CBL* gene in breast invasive carcinoma and a LINE-1 insertion into the first exon of the *BAALC* gene in a head and neck squamous cell carcinoma. Again, these findings are consistent with the protovirus hypothesis. However these tumors may also harbor mutations in “host” genes that regulate LINE-1 retrotransposition. A number of reviews were included in this topic that address recent studies on LINE-1 retrotransposition in cancer (Honda; Kemp and Longworth; Sciamanna et al.). In addition, identifying cellular genes and pathways that regulate LINE-1 transcription and activity is an active area of research, and two reviews discuss the current understanding regarding the regulation of LINE-1 retrotransposition in somatic cells, which may become dysregulated in cancer (Ariumi; Pizarro and Cristofari). The topic also includes two original research articles on the impact of endogenous retroviruses on genome evolution. In the article by Irie et al., the authors use dN/dS analysis and molecular approaches to validate their findings regarding the contribution of the *sushi-ichi* retrotransposon during the evolution of the zinc finger protein-encoding gene SIRH11/ZCCHC16 and the impact of this gene during eutherian brain evolution. In addition, another research article examines the evolution of the Tbx6 transcription binding sites, (ORRA1-ORRA1D), which are LTRs derived from the endogenous retroviruses, MaLRs (Yasuhiko et al.). The authors examine the impact on transcription of genes harboring these Tbx6 binding sites, using the Tbx6 knockout mouse. Their findings are coupled with biochemical and bioinformatic approaches. Finally two reviews nicely described the host cellular factors that impact the transcriptional dynamics of ERVs in the human genome (Buzdin et al.; Meyer et al.).

Overall the articles that were received for this topic: “Mobile Genetic Elements in Cellular Differentiation, Genome Stability, and Cancer” predominantly focus on the evolution of endogenous reverse transcriptases (RT), including the LINE-1 encoded RT, and the endogenous retroviruses ERVs and MaLR. These articles also summarize the findings in the field regarding these reverse transcriptases in normal biology and disease. These summaries and newly reported findings are consistent with the protovirus hypothesis (Temin, 1971; Shimotohno et al., 1980; Shimotohno and Temin, 1981). Identification of additional host factors and cellular pathways that contribute to LINE-1 retrotransposition will help further elucidate the protovirus hypothesis, as not all LINE-1 insertions occur in tumor suppressor or oncogenes. In addition, further studies regarding exogenous and endogenous reverse transcriptases will continue to shed light on the growing knowledge surrounding reverse transcription in the RNA world.

## Author contributions

All authors listed have made a substantial, direct and intellectual contribution to the work, and approved it for publication.

### Conflict of interest statement

The authors declare that the research was conducted in the absence of any commercial or financial relationships that could be construed as a potential conflict of interest.
